# 527. Burn Scar Thickness Predicts Dyschromia Presence and Delayed Resolution in a Black Patient Cohort

**DOI:** 10.1093/jbcr/irag033.224

**Published:** 2026-04-06

**Authors:** Seung W Chung, Bonnie C Carney

**Affiliations:** Georgetown University, Washington, DC; The Burn Center at MedStar Washington Hospital Center, Washington, DC

## Abstract

**Introduction:**

Post-burn dyschromia is highly prevalent in skin of color and leads to psychosocial complications for these patients. There are no treatments for this pathophysiology due to a limited understanding of its mechanism. Therefore here, potential drivers of hyperpigmentation were studied. It was hypothesized that dermal fibroblast-driven scar thickness is the dominant driver of dyschromia occurrence and persistence, outweighing burn size and acute critical illness.

**Methods:**

This was a retrospective analysis of Black patients (13.3%) in The Burn Model Systems Database (n = 2827) at discharge, 1-, 6-, 12-, and 18-months post-discharge. Variables were recorded as: scar thickness (yes/no), dyschromia (yes/no), %TBSA burned, and ventilator days (a proxy for acute critical illness). Cross-sectional analysis was performed at the discharge timepoint.

Associations were tested by Pearson correlations, Wilcoxon tests, and Fisher’s exact tests. Multivariable logistic regression (n = 142) modeled dyschromia with predictors of scar thickness %TBSA, ventilator days (≤4/>4 days). Effect modification by TBSA and ventilation was tested. Time-to-resolution of dyschromia was analyzed using Kaplan–Meier, Cox proportional hazards with age and sex, and the Weibull accelerated failure time (AFT) model.

**Results:**

At discharge, dyschromia and scar thickness were present in 163/177 (92%) and 95/161 (59%) subjects, respectively. Thickness correlated with dyschromia (r = 0.31). Thickness and dyschromia was strongly associated; Fisher OR = 21.0, 95% CI 2.95-914.9, *p*<.001. In logistic models, thickness remained the only independent predictor (OR = 22.0, 95% CI 3.9-418.3, *p*=.004), while %TBSA and ventilator days were not significant and no significant interactions between other variables were detected. Dyschromia resolved faster in thin scars (log-rank, *p*=.0068). In Cox models, thick scars had markedly slower resolution (HR = 0.05, *p*=.004); and the Weibull AFT estimated an acceleration factor of 24.3 (95% CI 1.75-337, *p*=.017), consistent in the direction and magnitude with the Cox model.

**Conclusions:**

Scar thickness, not burn size nor critical illness, dominates both the presence and persistence of dyschromia in this Black cohort, supporting a mechanistic model in which fibroblast-mediated dermal fibrosis affects epidermal pigment dysregulation.

**Applicability of Research to Practice:**

Scar treatments that modulate dermal fibrosis should be explored and include dyschromia resolution as a key endpoint, particularly for patients with skin of color.

**Funding for the study:**

This work was funded in part by award number KL2TR001432 from the National Center for Advancing Translational Science (NCATS/NIH).

